# Cost efficiency of calcium silicate and glass ionomer cements in pulpotomies of first mandibular primary molars

**DOI:** 10.4317/jced.63481

**Published:** 2026-08-28

**Authors:** Danilo Renato Valencia-Linares, Kilder Maynor Velasquez-Carhuapoma, Julissa Amparo Dulanto-Vargas, Kilder Maynor Carranza-Samanez

**Affiliations:** 1 School of Dentistry, Universidad Científica del Sur, Lima, Perú; 2 Research Group in Dental Sciences, Universidad Científica del Sur, Lima, Perú

**Keywords:** Biodentine, calcium silicate cements, glass ionomer cements, mineral trioxide aggregate, pulpotomy.

## Abstract

**Background:**

Calcium silicate cements (CSCs) and glass ionomer cements (GICs) are widely used in pulpotomy procedures because of their favorable biocompatibility, adhesive properties, and mechanical performance. However, limited evidence is available regarding the economic implications of their clinical use. This study evaluated the cost efficiency of CSCs and GICs used in pulpotomies of mandibular primary first molars (MPFMs).

**Materials and Methods:**

An in vitro experimental study was conducted using 72 MPFMs with standardized Class I cavities. The teeth were randomly allocated into six groups (n=12), combining two CSCs (mineral trioxide aggregate [MTA Angelus®] and Biodentine®) and three GICs (Vitremer®, Fuji IX® and Ketac Molar®). The cements were assessed according to commercial costs (per kit, per gram, and per dose), treatment-related costs (gross cost: material placed; actual cost: material prepared), and material utilization efficiency, defined as the gross-to-actual cost ratio. Data were analyzed using one-way ANOVA followed by Tukey’s post hoc test (p<0.05).

**Results:**

Among the CSCs, Biodentine showed the highest actual cost, whereas MTA Angelus showed the highest gross cost and greater utilization efficiency. Among the GICs, Vitremer presented the highest gross and actual costs, while Fuji IX and Ketac Molar demonstrated the highest utilization efficiency. The highest actual cost was observed for the Biodentine + Vitremer combination (USD 25.0), and the lowest for MTA Angelus + Fuji IX (USD 18.6). The highest efficiency ratios were found for MTA Angelus combined with Ketac Molar (53.36%) and with Vitremer (41.89%), whereas the remaining combinations showed values ≤25.62% (p<0.001).

**Conclusions:**

Significant differences in cost efficiency were identified among the materials evaluated. Overall, MTA Angelus combined with Ketac Molar demonstrated the greatest cost efficiency for pulpotomy procedures. Optimization of material presentations and dose sizes may reduce material waste and overall treatment costs.

## Introduction

Pulpotomy is a conventional treatment indicated for asymptomatic primary teeth or teeth with irreversible pulpitis caused by caries or trauma in the absence of radicular pathology [1,2]. This procedure involves the aseptic removal of the affected coronal pulp tissue [3,4]. Traditional approaches include hemostasis followed by the application of pulp-preserving medicaments such as formocresol, zinc oxide-eugenol, calcium hydroxide, and ferric sulfate [5,6]. Currently, bioactive calcium silicate-based cements, characterized by the release of calcium and hydroxyl ions, are widely used because of their long-term clinical success, biocompatibility, suitability for regenerative pulpal therapy, and minimally invasive properties [7-10].

Mineral trioxide aggregate (MTA), a Portland cement-derived material first introduced in 1993, is considered the first calcium silicate cement [11,12] and remains the gold standard for pulpotomy procedures [8,13]. Its mechanism of action involves the release of calcium hydroxide, which promotes reparative dentin formation and provides an effective seal of the pulp chamber [2]. Although MTA is biocompatible, non-cytotoxic, and bacteriostatic [11,14], its use may result in crown discoloration [8,15]. In addition, it has a prolonged setting time (>2 h) [16], high alkalinity (pH=12), and relatively high treatment costs [5].

Biodentine is a bioactive calcium silicate cement introduced in 2009 as an alternative to MTA [17]. Compared with MTA, it offers several biological advantages, including reduced tooth discoloration and faster calcium hydroxide release [4,18]. However, it demonstrates lower compressive strength and greater density and porosity [4,19]. Despite these limitations, Biodentine provides practical advantages, such as a shorter setting time and improved cost efficiency [10,20,21]. Recent systematic reviews have reported Biodentine to be either superior [10] or comparable [8,9] to MTA in terms of clinical outcomes for pulpal therapy in primary teeth. 

Adequate coronal restoration following pulpal therapy is essential to prevent microleakage and recurrent caries [2,3]. The progressive reduction in dental amalgam use following the 2017 Minamata Convention has shifted restorative strategies toward the preservation of dental structure and improved esthetics [22,23]. Glass ionomer cements are frequently used as bulk restorative materials [24]; however, their mechanical performance in deep carious lesions is inferior to that of composite resins and preformed crowns [25]. In contrast, resin-modified glass ionomer cements have demonstrated superior clinical performance in Class I and II cavities of teeth treated with pulpal therapy [24,25]. 

In Peru, the dental materials market relies predominantly on imported products, resulting in considerable price variability. Bioactive calcium silicate cements and esthetic glass ionomer cements are generally more expensive than traditional restorative materials. A previous *in vitro* study reported that Biodentine costs approximately 2.5 times more than MTA Angelus, whereas Ketac Molar is 1.8 times more expensive than Fuji IX for molar pulp chamber restorations [26]. 

Although most studies focus on the clinical success of contemporary pulpal materials, limited evidence is available regarding their cost and material efficiency during cavity preparation procedures. Therefore, this study aimed to evaluate the cost efficiency of calcium silicate and glass ionomer cements used in pulpotomy procedures of mandibular primary first molars. The null hypothesis was that there would be no statistically significant differences in cost efficiency among the cements evaluated.

## Materials and Methods

*Study design and ethical review*

This *in vitro* experimental study was conducted at the Stomatology Research Laboratory and approved by the Institutional Research Ethics Committee of the Universidad Científica del Sur in Lima, Peru (Approval No. 187-CIEI-CIENTÍFICA-2023). The protocol adhered to International Organization for Standardization (ISO) 6876:2012 standards and the CRIS guidelines (Checklist for Reporting *In-vitro* Studies).

*Sample selection*

The sample comprised 72 mandibular primary first molars indicated for extraction and donated by dental clinics in Lima, Peru. The teeth were randomly allocated into six groups (n=12 per group), corresponding to the combinations of two calcium silicate cements (MTA Angelus® and Biodentine®) and three glass ionomer-based cements: One light-cured resin-modified glass ionomer cements (Vitremer®) and two high-viscosity conventional glass ionomer cements (Fuji IX® and Ketac Molar®). Table 1 summarizes the characteristics of the materials evaluated, including classification, composition, manufacturer, batch number, total weight per kit, number of doses per kit, weight per dose, presentation, mixing time, and working time.

*Inclusion and exclusion criteria*

The inclusion criteria comprised mandibular primary first molars with no caries or non-extensive caries (International Caries Detection and Assessment System score <5; ≤40% structural loss), intact pulp chambers, a post-extraction period of ≤1 year, and storage in saline solution or distilled water. The exclusion criteria included teeth with irregular pulp chamber floors, non-uniform cement placement, or evidence of clumping or discoloration that could indicate indicate altered material properties.

*Sample size calculation*

The sample size was calculated using G*Power software (version 3.1.9.6), based on cost data obtained from a pilot study of 18 mandibular primary first molars (n=3 per group). A one-way ANOVA power analysis was conducted assuming a large effect size (f=0.8142), six groups, a standard deviation of 4.82, a confidence level of 99%, statistical power of 99%, and a 15% adjustment for potential losses. This resulted in a final sample size of 12 specimens per group.

*Pulpal chamber preparation*

The mandibular primary first molars were disinfected with 2.5% sodium hypochlorite for 5 min. All procedures were performed by a single calibrated operator. Access to the pulp chamber was obtained using a medium-grit, blue-ring, short-shank round diamond bur (MDT Micro Diamond, Israel). The cavity walls were refined using a medium-grit, red-ring, long-shank tapered diamond bur (MDT Micro Diamond, Israel). Preparations were performed with a push-button turbine (Gnatus, Brazil), under standardized conditions, with air pressure set at 0.18 MPa and water pressure at 0.198 MPa. The initial weight of each tooth was recorded in grams (g) using a digital scale (Aczet CY6102, USA).

*Pulpotomy cement preparation*

All cements were prepared according to the manufacturers’ instructions to ensure homogeneous mixing. The calcium silicate cements were placed using a Dycal carrier and condensed with a resin spatula to a standardized thickness of 2 mm from the pulp chamber floor, as measured with a North Carolina periodontal probe (Figure 1). The glass ionomer cements were applied 50 min after placement of the calcium silicate cement and condensed with a resin spatula. The Vitremer primer, gloss, and ionomer were light-cured using a 3M ESPE Elipar DeepCure-L LED curing unit. After removal of excess material, the teeth were weighed again to determine the post-preparation weight (Figure 2). 

*Cement sales and pulpotomy material costs*

The costs of the five cements were obtained in 2024 from official distributors or registered Peruvian dental suppliers across 25 regions of Peru, using information from websites, social media platforms, and professional referrals. Costs were estimated per kit, per gram, and per dose based on national averages. The pulpotomy material cost comprised gross costs, defined as the cement actually placed in the pulp cavity and actual costs were defined as the cement prepared according to the manufacturer’s instructions. The gross-to-actual cost ratio was used as an indicator of economic efficiency. Costs were analyzed for cements used alone and in combination. All values were recorded in Peruvian soles (PEN) and converted to US dollars (USD) using the exchange rate prevailing at the time of the study (USD 0.27 per PEN). The experimental workflow is illustrated in Figure 3.

*Data analysis*

Descriptive statistics included means, standard deviations, medians, interquartile ranges, frequencies, and percentages. Data normality was assessed using the Shapiro-Wilk test (n≤50) and the Kolmogorov-Smirnov test (n>50). When normality assumptions were met, inferential analyses were performed using independent t-tests (with unequal variances) and one-way ANOVA followed by Tukey’s or Tamhane’s post hoc tests. When normality assumptions were not met, the Mann-Whitney U test and the Kruskal-Wallis test with pairwise comparisons were applied. Statistical analyses were conducted using SPSS software version 27.0, and a *p-value* <0.05 was considered statistically significant.

## Results

*Sales costs*

Table 2 presents the 2024 sales costs per kit across Peruvian regions. The average sales costs (from highest to lowest) were as follows: Biodentine (USD 115.40 ± 7.52), followed by Vitremer (USD 53.37 ± 3.05), MTA Angelus (USD 35.85 ± 2.03), Ketac Molar (USD 32.26 ± 1.35), and Fuji IX (USD 30.68 ± 2.27). The regional prices of glass ionomer cements were similar (*p≥*0.057), whereas those of calcium silicate cements showed statistically significant variations (*p≤*0.001).

*Treatment costs*

Table 3 presents the costs of calcium silicate and glass ionomer cements used for pulpal therapy. Among the calcium silicate cements, Biodentine had the highest sales cost per kit and per dose, whereas MTA Angelus had the highest cost per gram. Among the glass ionomer cements, Vitremer showed the highest costs across all evaluated metrics (*p<*0.001). 

Among the calcium silicate cements, MTA Angelus exhibited the highest gross cost (USD 7.88), whereas Biodentine showed the highest actual cost (USD 23.08). In contrast, among the glass ionomer cements, Vitremer showed the highest gross and actual costs (USD 0.65 and USD 1.92, respectively), compared with Fuji IX (≤USD 0.45 and ≤USD 0.71, respectively) and Ketac Molar (≤USD 0.14 and ≤USD 0.23, respectively) (*p<*0.001). The gross-to-actual cost ratio was higher for MTA Angelus (40.22% ± 18.26%), Fuji IX (62.97% ± 14.64%), and Ketac Molar (62.71% ± 20.41%) than for Biodentine (10.93% ± 2.68%) and Vitremer (33.14% ± 14.37%) (*p<*0.001).

*Efficiency ratios*

Table 4 compares the combined costs of calcium silicate and glass ionomer cements. The gross cost was higher for MTA Angelus combined with any glass ionomer cement (USD 4.77-9.18) than for Biodentine combined with Fuji IX or Ketac Molar (USD 2.60-2.80) (*p<*0.001). The actual cost was highest for Biodentine + Vitremer (USD 25.0 ± 0.0) and lowest for MTA Angelus + Fuji IX (USD 18.6 ± 0.14) (*p<*0.001). The gross-to-actual cost ratio was highest for MTA Angelus combined with Ketac Molar (53.36% ± 21.32%) and with Vitremer (41.89% ± 9.36%), and lowest for Biodentine combined with any glass ionomer cement (11.17%-14.18%) (*p<*0.001).

## Discussion

Dental caries significantly affect the primary dentition and often triggers a cascade of events that may culminate in tooth loss [27]. Preventing caries in primary teeth helps avoid complex procedures and reduces the economic burden on families and healthcare systems [28]. Molars are among the teeth most frequently treated with pulpotomy procedures because of limited oral health awareness, inadequate hygiene habits, and restricted access to dental care [25]. This study evaluated various materials used in mandibular primary first molar pulpotomies and found that MTA Angelus and Ketac Molar were the most cost-efficient options, both individually and in combination.

The effectiveness of pediatric pulpal therapy materials has been extensively studied with respect to their properties, clinical performance, and treatment success [6,25]. Although evidence-based decision-making is recommended for pulpal therapy, cost considerations remain critical to addressing the socioeconomic needs of families [23]. This study demonstrates that acquisition and placement costs for mandibular primary first molar cavities vary according to whether calcium silicate cements and glass ionomer cements are used alone or in combination. These findings may assist clinicians in selecting more cost-efficient pulpal therapy strategies.

Sales costs per kit were generally consistent across the Peruvian regions. Biodentine and Vitremer were the most expensive materials. Price adjustments for imported brands, typically distributed through suppliers in Lima, are limited in regions with lower household incomes, particularly in the Amazon region, where material availability remains scarce despite tax incentives. Additionally, disparities in the procurement of calcium silicate cements are common because of their recognized clinical effectiveness in pulpal therapy [6].

The cost analysis of calcium silicate cements in mandibular primary first molars revealed higher actual costs for Biodentine than for MTA Angelus due to its higher weight per dose (0.7 g vs. 0.14 g). Conversely, the costs per gram was higher for MTA Angelus than for Biodentine (USD 128.02/g vs. USD 32.97/g). The gross-to-actual cost ratios was more favorable for MTA Angelus (≈40%) than for Biodentine (≈11%), indicating substantial material waste (>≈60%). Manufacturers, particularly those producing Biodentine, should consider reducing capsule doses to approximately 0.1 g for Class I cavities. The current packaging allows multiple pulpotomies per capsule; however, this scenario is uncommon in routine clinical practice [26].

Among the glass ionomer cements, Vitremer showed the highest gross and actual costs compared with Fuji IX and Ketac Molar. This difference is attributed to its larger per-dose spoon weight (0.09 g vs. 0.06-0.07 g) and higher powder-to-liquid ratio (2.0 g/mL vs. 1.25-1.47 g/mL). Fuji IX and Ketac Molar demonstrated a higher gross-to-actual cost ratio than Vitremer (≈63% vs. ≈34%), which corresponded to approximately 66% of material waste. Although the Vitremer technical datasheet recommends a 2:2 powder-to-liquid ratio, clinical practice in Class I cavities often favors a 1:1 adjustment.

The cost-benefit analysis of combinations of calcium silicate and glass ionomer cements revealed higher actual costs for Biodentine + Vitremer (USD 25) than for MTA Angelus + Fuji IX (USD 18.6), primarily due to differences in dosage and material composition [10,24]. Gross costs were higher for MTA Angelus combined with any glass ionomer cement (USD 4.77-10.81), reflecting its higher cost per gram. The highest efficiency was observed for MTA Angelus + Ketac Molar (53%) and MTA Angelus + Vitremer (42%), whereas all other combinations resulted in more than 74% of material waste.

Clinically, the creamy consistency of Biodentine’s facilitates handling but requires additional preparation time, as it must be mixed in an amalgamator. In contrast, MTA Angelus is easier to prepare but may generate more waste due to its granular texture [29]. All glass ionomer cements demonstrated satisfactory physical properties. However, the resin-modified formulation of Vitremer requires light curing, thereby prolonging the clinical procedure. Taken together with cost-benefit considerations, these factors may influence clinicians’ selection of the most appropriate material [26].

Consistent with previous literature, MTA Angelus combined with high-viscosity glass ionomer cements (Fuji IX or Ketac Molar) offers greater cost accessibility, whereas Biodentine and the resin-modified glass ionomer cement Vitremer provide improved handling and clinical performance. Nevertheless, clinicians must balance case-specific requirements with patient accessibility when selecting the most appropriate material [6,25]. Material availability is also influenced by taxation, geographic distribution, and the availability of pediatric dental specialists. Collaboration between manufacturers and clinicians is necessary to reduce material waste, thereby enhancing clinical efficiency and promoting environmental sustainability [30].

This study has several limitations. The cost and efficiency data in this study are specific to the Peruvian market in 2024, which limits the generalizability of the findings. Additional brands of calcium silicate and glass ionomer cements, as well as other cavity types (e.g., Class II), were not evaluated. Furthermore, the *in vitro* design did not account for clinical variables such as child cooperation, which may influence material consumption. Given the limited number of studies addressing the economic aspects of pulpal therapy materials, further research is warranted.

## Conclusions

This *in vitro* study demonstrates that calcium silicate cements and glass ionomer cements, used either alone or in combination, exhibit significant differences in sales costs and preparation and application costs when used for pulpotomies in mandibular primary first molars. MTA Angelus showed lower preparation costs and greater utilization efficiency compared with Biodentine in reparative pulpotomy treatments. High-viscosity glass ionomer cements (Fuji IX and Ketac Molar) presented higher preparation costs but demonstrated greater utilization efficiency than the resin-modified glass ionomer cement Vitremer in restorative procedures. Among the combinations evaluated, MTA Angelus and Ketac Molar demonstrated the highest overall cost efficiency. These findings suggest that optimizing commercial packaging formats and material proportions may improve cost efficiency in clinical practice. However, as the cost and efficiency data are specific to the Peruvian market in 2024, the generalizability of these results may be limited.

## Figures and Tables

**Table 1:** Table 1: Characteristics of the pulpal therapy cements used in the study.

**Description**	**MTA Angelus**	**Biodentine**	**Vitremer**	**Fuji IX**	**Ketac Molar**
Material classification	White calcium silicate cement	Calcium silicate cement	Light-cured resin-modified glass ionomer cement	Conventional glass ionomer cement	Conventional glass ionomer cement
Composition	Powder: Tricalcium silicate, dicalcium silicate, tricalcium aluminate, calcium oxide, tetracalcium aluminoferrite, and bismuth oxide.	Powder: Tricalcium silicate, zirconium oxide, calcium oxide, calcium carbonate, and pigments.	Powder: Radiopaque fluoroaluminosilicate glass.	Powder: Fluoroaluminosilicate glass and pigments.	Powder: Fluoroaluminosilicate glass, silicon dioxide, and pigments.
	Liquid: Distilled water.	Liquid: Calcium chloride solution and polycarboxylate.	Liquid: Modified polyalkenoic acid.	Liquid: Polyacrylic acid and water.	Liquid: Polyacrylic acid, itaconic acid, and water.
Manufacturer	Angelus®, Londrina, PR, Brazil	Septodont®, Saint-Maur-des-Fosses, France	3M ESPE®, St. Paul, MN, USA	GC corporation®, Itabashi-ku, Tokyo, Japan	3M® Deutschland GmbH, Neuss, Germany
Batch number	63953 / 69763	B29352	10076599	2211081	10075423
Total weight per kit	0.28 g	3.5 g	5 g	5 g	12.5 g
Number of doses per kit	2	5	≈55	≈83	≈178
Weight per dose	0.14 g	0.7 g	0.1 g	0.1 g	0.1 g
Presentation	Sachet (powder) + bottle (liquid)	Capsule (powder) and bottle (liquid)	Bottle (powder and liquid)	Bottle (powder and liquid)	Bottle (powder and liquid)
Supplied formulation	0.14 g powder	0.7 g powder	5 g powder	5 g powder	12.5 g powder
	3 ml distilled water	0.20 ml liquid	2.5 ml liquid	4 ml liquid	8.5 ml liquid
			2 ml primer	1 measuring spoon. (≈0.06 g)	1 measuring spoon. (≈0.07 g)
			2 ml gloss liquid		
			1 measuring spoon (≈0.09 g)		
Manufacturer-recommended handling protocol	Mix 0.14 g of powder with one drop of liquid component on a glass slab. Continue mixing until a homogeneous consistency is achieved.	Shake the capsule (0.7 g). Add five drops of liquid component. Triturate in an amalgamator for 30 s at 4000 rpm.	Apply the primer. Gently air-dry for 5 s, and light-cure for 20 s. Mix the powder and liquid at a 2:2 ratio. Load the mixture into a Centrix syringe and apply to the prepared cavity. Light-cure for 40 s. Finally, apply a gloss coating and light-cure for an additional 20 s.	Condition the cavity with one drop of liquid component. Rinse for 10 s and gently air-dry for 5 s using a three-way syringe. Mix the powder and liquid at a 1:1 ratio and apply immediately to the prepared cavity.	Condition the cavity with one drop of liquid. Rinse for 10 s and gently air-dry for 5 s using a three-way syringe. Mix the powder and liquid at a 1:1 ratio and apply immediately to the prepared cavity.
Mixing time	30 s	30 s	30 s	30 s	45 s
Working time	2-3 h	12 min	2 min	2 min	5 min

**Table 2:** Table 2: Sales costs (USD) of pulpal therapy cements per kit across Peruvian regions in 2024.

**Region**	**n**	Calcium Silicate Cement*				Glass Ionomer Cement*					
		**MTA Angelus**		**Biodentine**		**Vitremer**		**Fuji IX**		**Ketac Molar**	
		**Mean**	**SD**	**Mean**	**SD**	**Mean**	**SD**	**Mean**	**SD**	**Mean**	**SD**
Total	3	35.85	2.03	115.4	7.52	53.37	3.05	30.68	2.27	32.26	1.35
Amazonas	3	34.84ab	1.07	114.06ab	2.30	54.82	1.53	31.39	0.93	32.27	0.77
Ancash	3	35.37ab	0.77	105.22ab	1.53	51.99	1.40	31.12	1.23	31.74	0.15
Apurímac	3	34.92ab	2.76	125.99ab	5.78	50.75	0.85	28.21	1.46	30.06	0.77
Arequipa	3	38.02ab	0.77	118.92ab	0.77	56.59	1.53	35.01	0.80	33.69	0.46
Ayacucho	3	31.39b	0.77	119.36ab	0.00	52.17	3.06	28.56	0.67	32.27	0.77
Callao	3	35.72ab	2.38	106.98ab	1.53	52.17	1.53	30.24	1.40	31.83	0.00
Cajamarca	3	37.58ab	0.77	121.57ab	3.83	52.17	1.53	31.21	0.61	33.16	0.00
Cuzco	3	34.92ab	2.03	122.90ab	6.13	62.95	0.55	30.24	0.46	33.60	0.77
Huancavelica	3	37.14ab	0.00	119.36ab	0.00	50.40	0.00	31.83	0.00	31.74	0.15
Huánuco	3	37.58ab	0.77	119.36ab	0.00	51.28	1.53	32.18	0.31	33.51	0.61
Ica	3	36.25ab	0.77	110.52ab	3.83	52.17	1.53	34.48	0.00	33.60	0.77
Junín	3	36.52ab	0.67	106.98ab	1.53	54.82	1.53	32.89	1.40	33.60	0.77
La Libertad	3	35.37ab	1.53	119.36ab	0.00	53.05	0.00	29.71	0.80	31.83	0.00
Lambayeque	3	36.25ab	0.77	110.52ab	3.83	52.17	1.53	28.65	0.70	32.71	0.77
Lima	3	37.49ab	0.85	103.89ab	3.83	49.96	0.77	31.12	1.07	34.31	0.31
Loreto	3	32.27ab	0.77	106.10ab	11.49	53.93	1.53	28.91	0.96	31.30	0.70
Madre de Dios	3	37.14ab	0.00	125.99a	0.00	53.93	1.53	31.74	0.15	31.83	0.00
Moquegua	3	31.83ab	0.00	119.36ab	0.00	58.36	0.00	31.83	0.00	31.83	0.00
Pasco	3	34.66ab	0.31	118.48ab	1.53	52.61	3.34	26.88	1.88	30.50	0.00
Piura	3	36.69ab	0.77	103.45ba	0.00	51.28	1.53	27.59	1.84	30.95	0.77
Puno	3	35.99ab	1.36	121.57ab	3.83	51.28	1.53	30.59	0.93	30.50	1.33
San Martin	3	37.14ab	1.33	117.15ab	3.83	56.15	0.77	34.48	0.00	31.39	0.77
Tacna	3	38.46a	0.00	106.10ab	0.00	53.05	0.00	31.12	1.34	32.71	0.77
Tumbes	3	36.12ab	1.46	119.36ab	0.00	52.79	2.71	28.69	1.76	32.94	2.43
Ucayali	3	36.25ab	1.53	118.48ab	1.53	53.93	0.77	30.24	0.46	31.83	0.00
*p* *value*		0.001**		<0.001**		≥0.059		≥0.061		≥0.057	

n: Number of specimens; SD: Standard deviation. *Cost corresponds to the presentation specified in Table 1. Different lowercase letters indicate statistically significant differences among columns (Kruskal-Wallis test with pairwise post hoc comparisons

**Table 3:** Table 3: Costs of pulpotomy treatments according to the use of calcium silicate or glass ionomer cement alone.

**Characteristics**	**Calcium Silicate Cement**					**Glass Ionomer Cement**						
	**MTA Angelus**		**Biodentine**		*P* ** value†**	**Vitremer**		**Fuji IX**		**Ketac Molar**		*p* ** value‡**
	**Mean ± SD**	**Median [IQR]**	**Mean ± SD**	**Median [IQR]**		**Mean ± SD**	**Median [IQR]**	**Mean ± SD**	**Median [IQR]**	**Mean ± SD**	**Median [IQR]**	
Cost per kit (USD)	35.85 ± 2.04	36.08 [34.48-37.14]b	115.40 ± 7.51	119.36 [106.1-119.36]a	<0.001*	53.37 ± 3.05	53.05 [50.4-55.7]a	30.68 ± 2.27	30.5 [29.18-31.83]c	32.26 ± 1.35	31.83 [31.83-33.16]b	<0.001*
Cost per gram (USD)	128.02 ± 7.27	128.84 [123.15-132.63]a	32.97 ± 2.15	34.1 [30.31-34.1]b	<0.001*	10.67 ± 0.61	10.61 [10.08-11.14]a	6.14 ± 0.45	6.1 [5.84-6.37]b	2.58 ± 0.11	2.55 [2.55-2.65]c	<0.001*
Cost per dose (USD)	17.92 ± 1.02	18.04 [17.24-18.57]b	23.08 ± 1.50	23.87 [21.22-23.87]a	<0.001*	0.96 ± 0.05	0.95 [0.91-1]a	0.37 ± 0.03	0.37 [0.35-0.38]b	0.18 ± 0.01	0.18 [0.18-0.19]c	<0.001*
Gross weight (g)	0.06 ± 0.04B	0.06 [0.04-0.07]	0.08 ± 0.02A	0.07 [0.07-0.09]	0.047*	0.06 ± 0.03 AB	0.06 [0.05-0.07]	0.07 ± 0.03A	0.07 [0.06-0.09]	0.05 ± 0.02B	0.05 [0.04-0.07]	0.024*
Gross cost (USD)	7.88 ± 5.09A	7.07 [4.76-9.29]	2.52 ± 0.62B	2.45 [2.17-2.94]	<0.001*	0.65 ± 0.28A	0.64 [0.5-0.78]	0.45 ± 0.16B	0.42 [0.39-0.55]	0.14 ± 0.06C	0.13 [0.1-0.18]	<0.001*
Net weight (g)	0.15 ± 0.03B	0.14 [0.14-0.14]	0.70 ± 0A	0.70 [0.70-0.70]	<0.001*	0.18 ± 0A	0.18 [0.18-0.18]	0.12 ± 0.02B	0.12 [0.12-0.12]	0.09 ± 0.03C	0.07 [0.07-0.11]	<0.001*
Actual cost (USD)	18.92 ± 4.16B	17.92 [17.92-17.92]	23.08 ± 0A	23.08 [23.08-23.08]	<0.001*	1.92 ± 0A	1.92 [1.92-1.92]	0.71 ± 0.15B	0.74 [0.74-0.74]	0.23 ± 0.08C	0.18 [0.18-0.27]	<0.001*
Gross-to-actual cost ratio (%)	40.22 ± 18.26A	39.46 [26.57-51.86]	10.93 ± 2.68B	10.61 [9.41-12.73]	<0.001*	33.8 ± 14.37B	33.14 [26.03-40.67]	62.97 ± 14.64A	57.5 [53.13-76.21]	62.71 ± 20.41A	67.07 [48.89-74.21]	<0.001*

SD: Standard deviation. IQR: Interquartile range.

**Table 4:** Table 4: Costs of pulpotomy treatments according to combinations of calcium silicate and glass ionomer cements.

**Characteristics**	**MTA Angelus + Vitremer**		**MTA Angelus + Fuji IX**		**MTA Angelus + Ketac Molar**		**Biodentine + Vitremer**		**Biodentine + Fuji IX**		**Biodentine + Ketac Molar**		*p* ** value**
	**Mean ± SD**	**Median [IQR]**	**Mean ± SD**	**Median [IQR]**	**Mean ± SD**	**Median [IQR]**	**Mean ± SD**	**Median [IQR]**	**Mean ± SD**	**Median [IQR]**	**Mean ± SD**	**Median [IQR]**	
Gross weight (g)	0.12 ± 0.03AB	0.12 [0.11-0.13]	0.1 ± 0.03B	0.1 [0.09-0.12]	0.13 ± 0.07AB	0.13 [0.1-0.14]	0.15 ± 0.04A	0.15 [0.13-0.18]	0.15 ± 0.04A	0.15 [0.13-0.18]	0.13 ± 0.02AB	0.13 [0.12-0.15]	0.024*
Gross cost (USD)	9.18 ± 4.29A	8.19 [6.98-9.6]	4.77 ± 1.01A	4.75 [4.17-5.41]	10.81 ± 6.33A	9.45 [8.69-11.27]	3.55 ± 0.75B	3.53 [2.96-3.86]	2.8 ± 0.81C	2.72 [2.39-3.17]	2.6 ± 0.44C	2.64 [2.28-2.73]	<0.001*
Net weight (g)	0.33 ± 0.04C	0.32 [0.32-0.32]	0.25 ± 0.02D	0.26 [0.26-0.26]	0.23 ± 0.05D	0.21 [0.21-0.25]	0.88 ± 0A	0.88 [0.88-0.88]	0.82 ± 0.03B	0.82 [0.82-0.82]	0.79 ± 0.03B	0.77 [0.77-0.84]	<0.001*
Actual cost (USD)	21.34 ± 5.17ABC	19.84 [19.84-19.84]	18.6 ± 0.14D	18.66 [18.66-18.66]	19.63 ± 5.16ABC	18.1 [18.1-18.19]	25 ± 0A	25 [25-25]	23.82 ± 0.16B	23.82 [23.82-23.82]	23.32 ± 0.09C	23.26 [23.26-23.44]	<0.001*
Gross-to-actual cost ratio (%)	41.89 ± 9.36A	41.28 [35.18-48.39]	25.62 ± 5.36B	25.46 [22.57-29.02]	53.36 ± 21.32A	52.18 [47.78-62.24]	14.18 ± 2.98C	14.11 [11.85-15.46]	11.76 ± 3.39C	11.4 [10.04-13.29]	11.17 ± 1.9C	11.35 [9.78-11.68]	<0.001*
Calcium silicate cement (%)	93.14 ± 3.21B	93.07 [92.19-94.16]	91.18 ± 3.29B	91.56 [88.62-92.88]	98.59 ± 0.7A	98.76 [98.35-99.09]	79.59 ± 5.96C	79.63 [75.98-83.87]	81.89 ± 5.87C	81.87 [78.89-85.45]	94.11 ± 2.21B	94.27 [92.29-95.71]	<0.001*
Glass ionomer cement (%)	6.86 ± 3.21B	6.93 [5.84-7.81]	8.82 ± 3.29B	8.44 [7.12-11.38]	1.41 ± 0.7C	1.24 [0.91-1.65]	20.41 ± 5.96A	20.37 [16.13-24.02]	18.11 ± 5.87A	18.13 [14.55-21.11]	5.89 ± 2.21B	5.73 [4.29-7.71]	<0.001*

SD, standard deviation. IQR, interquartile range.

**Figure 1:**
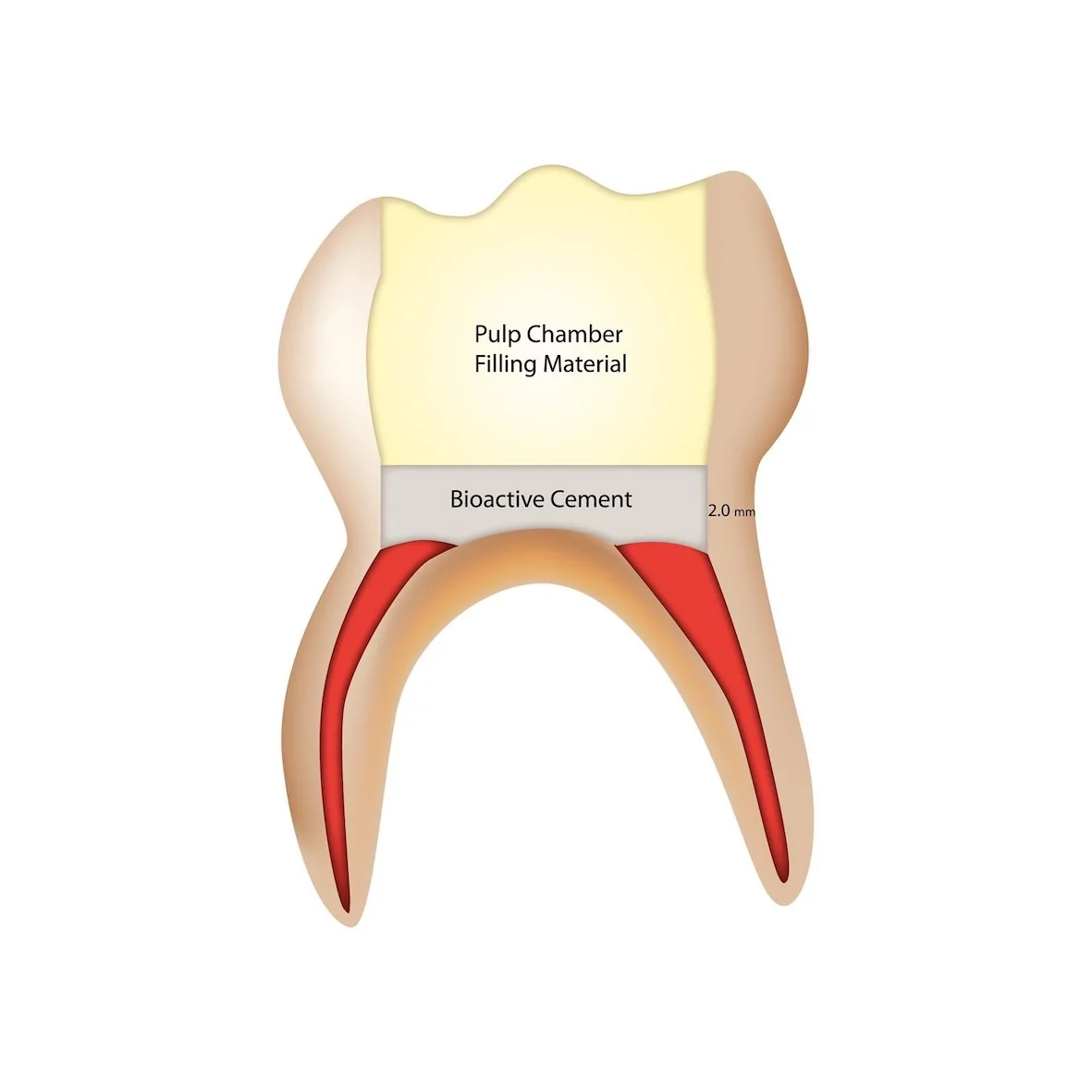
Figure 1. Application protocol of pulpal therapy cements in mandibular primary first molars.

**Figure 2:**
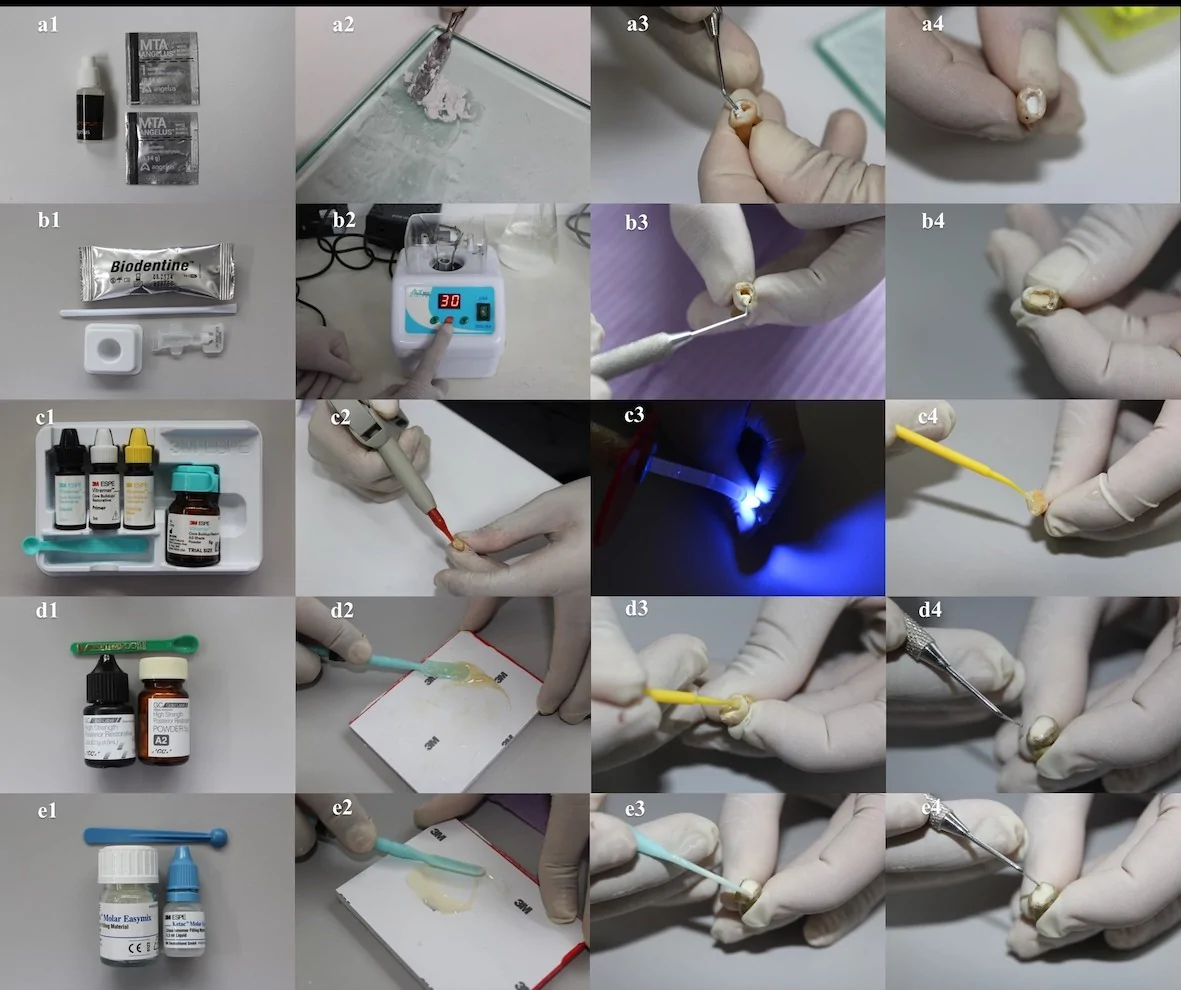
Figure 2. Representative photographs of the cement (1), preparation (2), application (3), and treated tooth (4) for MTA Angelus (a), Biodentine (b), Vitremer (c), Fuji IX (d), and Ketac Molar (e).

**Figure 3:**
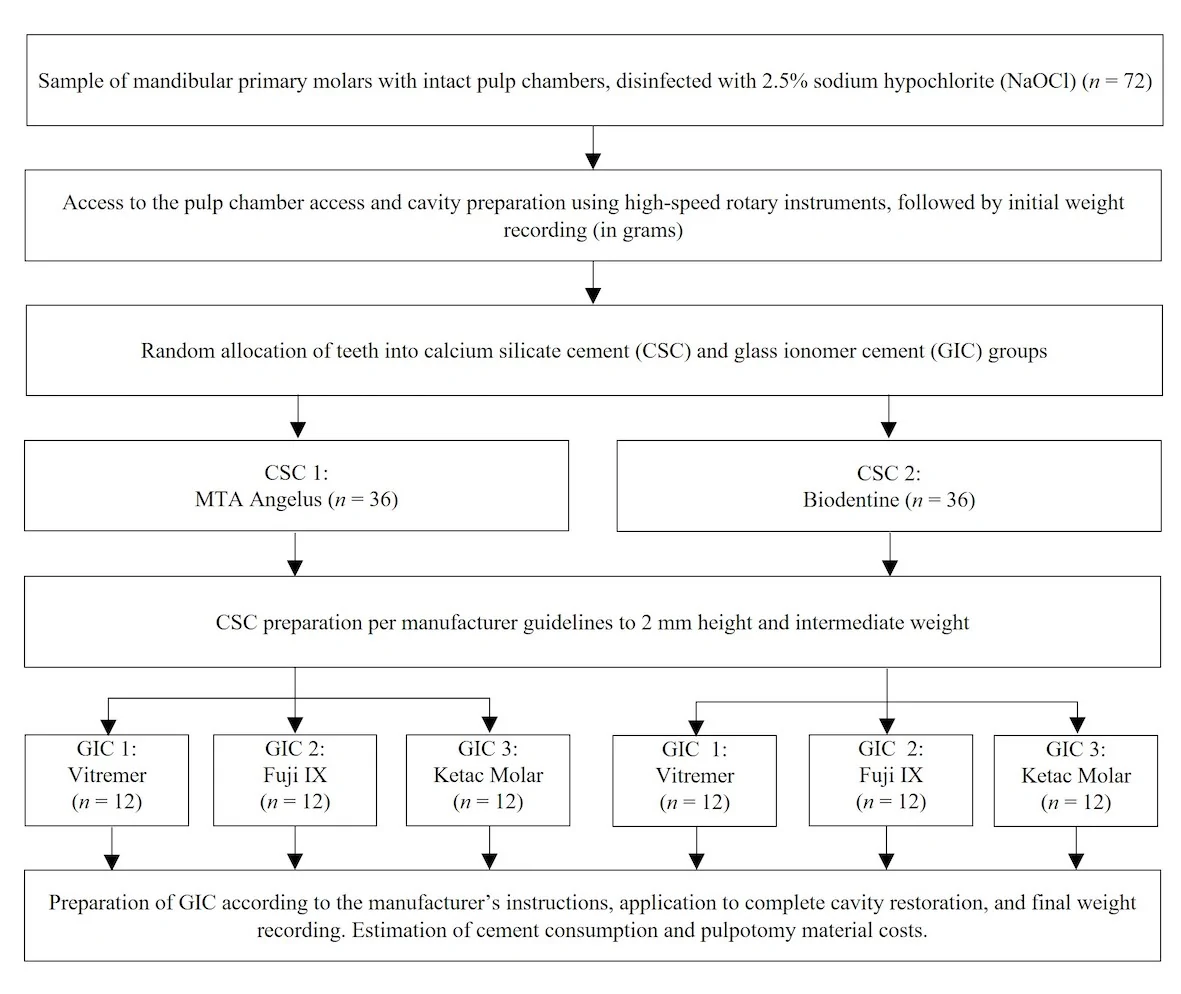
Figure 3. Flowchart of the experimental procedure for the application of calcium silicate and glass ionomer cements.

## Data Availability

The datasets used and/or analyzed during the current study are available from the corresponding author.
